# A Rare Case of *Strongyloides stercoralis* Hyperinfection in a Diabetic Patient from Romania—Case Report and Review of the Literature

**DOI:** 10.3390/pathogens12040530

**Published:** 2023-03-29

**Authors:** Carmen Costache, Ioana Alina Colosi, Vlad Sever Neculicioiu, Diana Ioana Florian, Bobe Petrushev, Alexandra Vasvari, Andrada Seicean

**Affiliations:** 1 Department of Microbiology, “Iuliu Hatieganu” University of Medicine and Pharmacy, 400012 Cluj-Napoca, Romania; 2 Regional Institute of Gastroenterology and Hepatology, 400162 Cluj-Napoca, Romania; 3 Department of Gastroenterology, “Iuliu Hatieganu” University of Medicine and Pharmacy, 400012 Cluj-Napoca, Romania

**Keywords:** *Strongyloides*, strongyloidiasis, hyperinfection, diabetes, gastric, intestinal obstruction, immunosuppressed, immunocompetent

## Abstract

Severe cases of strongyloidiasis are most often associated with multiple causes of immune suppression, such as corticoid treatment and HTLV (human T-lymphotropic virus) coinfection. Diabetes is not traditionally considered a risk factor for the development of severe strongyloidiasis. We report a rare case of autochthonous severe strongyloidiasis in Romania, a European country with a temperate climate. A 71-year-old patient with no prior travel history was admitted with multiple gastrointestinal complaints and recent weight loss. CT (computed tomography) scans indicated duodenal wall thickening, and duodenal endoscopy evidenced mucosal inflammation, ulcerations and partial duodenal obstruction at D4. Microscopic examination of stool samples and biopsy specimens from the gastric and duodenal mucosa revealed an increased larval burden characteristic of *Strongyloides stercoralis* hyperinfection. Sequential treatment with albendazole and ivermectin achieved parasitological cure and complete recovery. The novelty of our case stems from the scarcity of severe strongyloidiasis cases reported in Europe and especially in Romania, the absence of other risk factors in our patient aside from diabetes, the involvement of the gastric mucosa and the rare presentation as partial duodenal obstruction. This case highlights the importance of considering strongyloidiasis as a differential diagnosis, even in temperate climates where cases are sporadic, in cases in which immune suppression is not evident and in the absence of eosinophilia. The case is presented in the context of the first literature review examining the relationship between severe strongyloidiasis and diabetes, emphasizing diabetes as a possible risk factor for severe strongyloidiasis.

## 1. Introduction

*Strongyloides stercoralis*, also known as threadworm, is a common roundworm that causes infections in humans mainly through soil transmission. Even though the first record of this parasite was established in the late 19th century in a fecal sample from a French soldier in Vietnam [1,2], the details of its life cycle, pathology and clinical aspects of human strongyloidiasis were documented half a century later in the 1930s [3,4].

*S. stercoralis* is an exceptional helminthic parasite with a complex life cycle that includes free-living, parasitic and autoinfective cycles [5]. Strongyloidiasis is frequently a chronic infection, maintained by the endogenous autoinfective cycle. Severe forms of strongyloidiasis are seen in immunosuppressed individuals as hyperinfection syndrome or disseminated strongyloidiasis. In individuals with impaired cell-mediated immunity, the hyperinfection syndrome may be fatal [3,6,7]. 

Intestinal strongyloidiasis may present, as many other parasitic infections, with a wide spectrum of manifestations, ranging from asymptomatic to unspecific gastrointestinal symptoms, such as diarrhea, constipation, anorexia, abdominal discomfort or pain and urticaria [8,9,10].

The terms hyperinfection syndrome and disseminated strongyloidiasis are sometimes used interchangeably in the literature. Hyperinfection syndrome can be defined as a substantial increase in larval multiplication and parasitic burden, manifested as gastrointestinal (intestinal obstruction, peritonitis, gastrointestinal bleeding) or respiratory symptoms (pneumonitis, respiratory failure). Dissemination of larvae outside of the gastrointestinal and pulmonary tracts defines disseminated strongyloidiasis and is usually accompanied by meningitis, encephalitis or bloodstream infections due to Gram-negative enteric bacteria [10,11,12].

Though frequently asymptomatic, chronic infections may be dangerous if left undiagnosed, because they may switch to life-threatening hyperinfection or disseminated forms in the case of immunosuppressive therapy with corticosteroids [10].

We report an unusual case of partial duodenal obstruction caused by *S. stercoralis* hyperinfection in a patient with type 2 diabetes. The rarity of our case stems from the scarcity of severe strongyloidiasis cases in Europe, the absence of other immune suppression factors aside from diabetes in our patient, the involvement of the gastric mucosa and the presentation as partial intestinal obstruction. 

Due to the rarity of the presentation, we performed a review of the literature in which we examined the extent to which diabetes can be considered a risk factor for the development of severe strongyloidiasis.

## 2. Case Report

### 2.1. Presentation and Medical History

A 71-year-old patient presented to the emergency department of the Regional Institute of Gastroenterology and Hepatology, Cluj-Napoca, Romania, with multiple gastrointestinal complaints: anorexia, vomiting, recent weight loss (8 kg/17.64 pounds), epigastric pain and fatigue. The symptoms first appeared a month earlier but worsened two weeks prior to admission. 

The patient’s medical history included type II diabetes, stage III hypertension, benign prostatic hyperplasia, colonic diverticular uncomplicated disease and no prior surgery. His medication consisted of metoprolol, indapamide, insulin (long acting) and quinapril. The patient reported no history of tobacco use and low to moderate alcohol intake (8 units/week). He lived in a rural area, had contact with domestic animals, occasionally worked in agriculture and never traveled abroad. The patient reported no recent changes in bowel movements.

### 2.2. Physical Examination, Blood Tests, Ultrasound and CT Scan

The initial examination evidenced no fever, a normal cardiopulmonary examination and tenderness in the epigastric area. White cell count was elevated (16.600 × 10^3^ µL) with neutrophilia, the absence of eosinophilia, an increased CRP (C-reactive protein) level (16.1 mg/dL), a normal procalcitonin level and negative blood and urine bacterial cultures.

Abdominal ultrasound revealed a distended stomach with significant stasis, enlarged mesenteric lymph nodes, colonic wall thickness and distended small bowels. 

**Figure 1:**
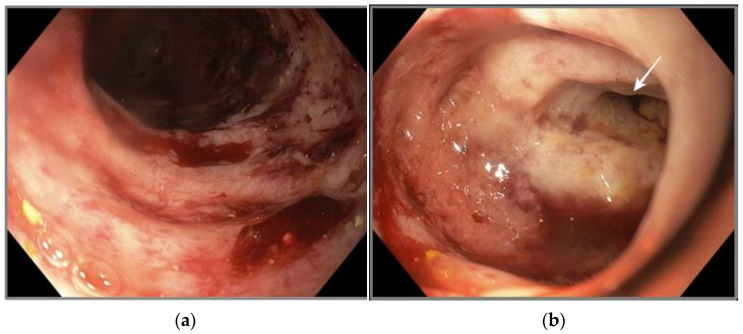
Gastroscopy—duodenum. (**a**) Oedema and erosions of the duodenal mucosa; (**b**) partial duodenal stenosis at D4 level (white arrow).

According to these findings, the decision was made to perform a gastroscopy, which revealed inflammation and acute ulcerations in the duodenal mucosa and a partial circumferential stenosis at the D4 level. Multiple biopsy specimens were taken from the gastric and duodenal mucosa. The aspect of the duodenum during gastroscopy is presented in Figure 1.

**Figure 2:**
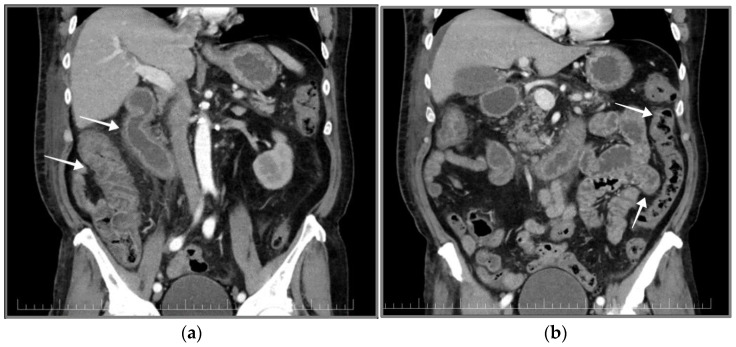
Contrast CT scan of the thorax, abdomen and pelvic region. (**a**) Ascending colon and duodenum with contrast-enhanced thickened wall (white arrows); (**b**) small bowel and descending colon with normal aspect (white arrows).

Consecutively, a CT scan of the thorax, abdomen and pelvis was conducted. The CT scan revealed duodenal wall thickening with contrast enhancement of an inflammatory pattern, lymph nodes under 10 mm and infiltration of the periduodenal fat tissue. A similar aspect was described in the colon along with a mild accumulation of ascites (Figure 2).

Due to the inflammatory aspect of the gastrointestinal tract, an infection was assumed, and the following tests were performed, yielding negative results: *Clostridium difficile* rapid stool test, stool culture for *Shigella* spp., *Salmonella* spp. and *Yersinia* spp. 

### 2.3. Stool Examination

An O&P (ova and parasites) examination was performed with a Zeiss microscope (Jena, Germany) using 10× and 40× objectives (100× and 400× magnification) and wet mounts from fresh stool stained with Lugol’s iodine.

The macroscopic examination revealed a bloody mucous aspect of the stool. Microscopic examination revealed parasitic structures, which were still alive and moving at the moment of examination. Based on the rhabditiform morphology of the esophagus, the length of approximately 250 μm, short buccal cavity and prominent genital primordium, they were identified as rhabditiform larvae of *Strongyloides stercoralis*.

**Figure 3:**
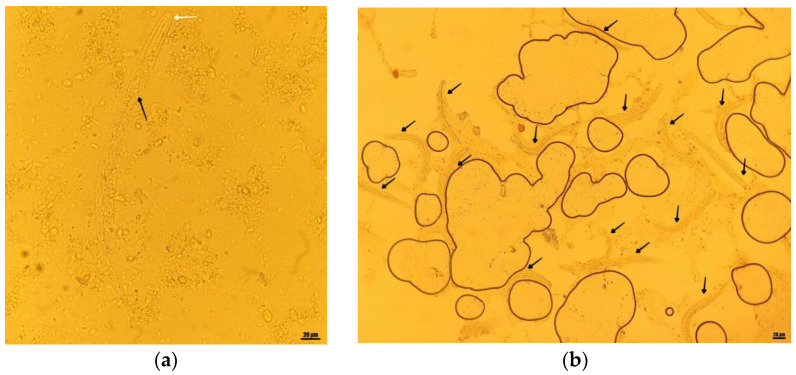
O&P examination—light microscopy wet mounts. (**a**) Rhabditiform *S. stercoralis* larvae—short buccal cavity (white arrow), rhabditiform esophagus (black arrow); Lugol’s iodine-stained wet mount, 40×; (**b**) rhabditiform *S. stercoralis* larvae (black arrows), 10–15/microscopic field; Lugol’s iodine-stained wet mount, 10×.

Considering the increased numbers of larval structures in most microscopic fields (10–15 larvae/field), we interpreted these findings as an increased parasitic burden, characteristic for hyperinfection syndrome (Figure 3).

### 2.4. Gastric and Duodenal Histology

**Figure 4:**
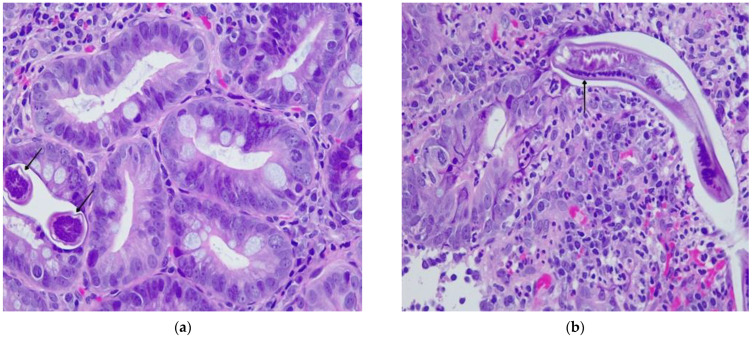
Duodenal histopathology. (**a**,**b**) *S. stercoralis* larvae—cross and longitudinal sections in the duodenal mucosa (black arrows); mixed-cell inflammatory infiltrate in the surface epithelium, edema and microhemorrhages in the lamina propria; light microscopy; Hematoxylin–Eosin stain 100×.

A histopathologic examination of the gastric and duodenal biopsy further confirmed the presence of *S. stercoralis* larvae. Histopathology from the gastric antral and duodenal mucosa revealed an abundant mixed cell inflammatory infiltrate in the surface epithelium, edema and microhemorrhages in the lamina propria (Figure 4). 

### 2.5. Diagnosis, Treatment and Additional Examinations

The presence of *S. stercoralis* larvae was evidenced in biopsies from the gastric antral and duodenal mucosa and in wet mounts from fresh stool samples. Identification of larvae was performed according to morphologic criteria. Due to the beforementioned criteria, molecular diagnosis was not deemed necessary for identification. No involvement of other body sites was evidenced. The final diagnosis was concluded as partial duodenal stenosis due to *S. stercoralis* hyperinfection in a patient with probable immunosuppression determined by diabetes, as per the definition provided by the CDC (Centers for Disease Control and Prevention, Atlanta, GA, USA) for hyperinfection syndrome [12]. 

Following the diagnosis, the patient was transferred to the Infectious Disease Hospital in Cluj-Napoca, Romania and received 10 days of albendazole 400 mg twice daily, followed by 200 mcg/kg ivermectin for three days. 

In order to further investigate a possible source of immune suppression, antibody tests were performed for HIV (human immunodeficiency virus) and HTLV 1/2, with negative results.

The patient’s evolution was positive under treatment, with negative O&P stool exams at the end of treatment and at a 2-week follow-up. A further follow-up gastroscopy was performed 2 months after the discharge, revealing a normal duodenum, with a slightly erythematous mucosa and no evidence of larvae in stool.

## 3. Discussion

Although encountered worldwide, infections with *S. stercoralis* are more common in developing countries and mostly in tropical and subtropical climates. However, strongyloidiasis can also be found in temperate climates [13]. Recent statistical models estimate the prevalence of this infection at around 600 million cases worldwide [14]. Strongyloidiasis is a sporadic disease in developed countries, where fecal contamination of the soil is rare. In developed countries, strongyloidiasis is more frequent in rural areas and predominantly affects individuals working in farming and mining activities. 

Limited data from multiple EU (European Union) countries reported by the ECDC (European Centre for Disease Prevention and Control) estimate the prevalence of rates of *S. stercoralis* infections between 3.3% and 5.6% in Italy, Spain and France [15]. At the time of writing, limited data are available regarding the prevalence of strongyloidiasis in Romania. Despite the temperate and continental climate, some historical foci of endemic strongyloidiasis have been described in Romania, mainly in rural underdeveloped areas [16]. Even though most available epidemiological data stem from studies published in the mid and late 20th century, a recent review established the historical prevalence of *S. stercoralis* between 0.01–16%, with higher values being reported in select cases of institutionalized children 13.4–30% [17]. However, at the time of writing, strongyloidiasis can be considered at most sporadic in Romania, with only three published case reports available [18,19,20].

Severe strongyloidiasis (hyperinfection or disseminated disease) is relatively rarely reported in Europe, with a recent systematic review identifying 50 cases of autochthonous infection [21]. To our knowledge, only two other cases of *S. stercoralis* hyperinfection or disseminated disease have been published in Romania, both featuring patients with altered immune status due to corticoid therapy and kidney disease or a combination of alcoholic liver disease and acute pancreatitis [19,20]. 

Human contamination is mainly realized transcutaneously through contact with fecal-contaminated soil [3], which seems the most probable transmission route in the case of our patient.

Infections with *S. stercoralis* are often difficult to diagnose, particularly in the case of severe strongyloidiasis, in which the diagnosis is frequently delayed due to the unspecific clinical presentation, shortcomings of diagnostic techniques [22,23] and a low index of clinical suspicion [24]. Multiple diagnostic procedures are available, including direct copro-diagnostic procedures (stool wet mounts and concentration techniques such as formalin–ether, Baermann, Harada–Mori and agar plate methods), serologic assays, molecular assays and complementary techniques such as endoscopy, histological examinations and imaging techniques [25,26,27,28,29]. Direct stool examinations are considered the gold standard in the diagnosis of strongyloidiasis, despite the lower sensitivity compared to serologic techniques [22]. 

Eosinophilia is present in up to 75% of individuals with chronic intestinal strongyloidiasis [12], but is frequently absent in severe cases of hyperinfection or dissemination [30,31,32]. In our case, the patient’s eosinophil count was normal.

Intestinal obstruction is a rare complication of strongyloidiasis, with only a few cases being reported in the literature [33]. Our patient presented with a partial circumferential duodenal obstruction at D4, with a complete remission at two months follow-up after treatment with albendazole and ivermectin.

Gastric involvement in strongyloidiasis has been rarely reported and in most cases involved immunosuppressed patients. According to a recent review, only seven cases of gastric involvement have been reported in immunocompetent patients [34].

Severe strongyloidiasis can be often fatal, with a reported mortality rate of ~60% [30,32,35]. Ivermectin is currently considered the best treatment option for strongyloidiasis [36,37], but albendazole and its derivates can be taken into consideration as suitable alternatives in the absence of ivermectin. In the case of hyperinfection syndrome or disseminated strongyloidiasis, the CDC currently recommends a prolonged treatment regimen with ivermectin at 200 µg/kg per day, until stool or other samples are negative for 2 weeks [12]. Our patient was treated for ten days with 400 mg albendazole twice daily, due to the temporary unavailability of ivermectin. After ivermectin became available, the treatment was prolonged for three more days. The combined treatment of albendazole and ivermectin achieved a parasitological cure, as evidenced by the follow-up exams at discharge and two months later.

The most well-known risk factors for *S. stercoralis* hyperinfection or disseminated disease are coinfection with HTLV-1 and corticosteroid use [30]. As summarized by recent reviews, multiple other risk factors have also been associated with severe strongyloidiasis: immunosuppressive drugs, anti-neoplastic agents, monoclonal antibodies, total body irradiation, hypogammaglobulinemia (associated with nephrotic syndrome and multiple myeloma), hematologic malignancies, solid organ transplant, hematopoietic stem cell transplant, HIV and immune reconstitution inflammatory syndrome, alcoholism and malnutrition [10,38,39,40]. 

Our patient did not present any discernable causes of immune suppression, with the exception of diabetes. Immune suppression and diabetes are well-known risk factors for multiple parasitic diseases caused by helminths and protozoa [41,42,43]. Despite this fact, the relationship between strongyloidiasis and diabetes is not completely understood, with evidence pointing both towards [44,45] and against [46,47] an association between these two diseases. Additionally, diabetes has been highlighted as a risk factor for treatment failure in intestinal strongyloidiasis [48]. Furthermore, the extent to which diabetes can be considered a risk factor for severe strongyloidiasis is also unclear. 

## 4. Literature Review

### 4.1. Review Methodology

We performed a review of the literature regarding the relationship between severe strongyloidiasis and diabetes. The search was performed up to 15 March 2023 in four databases: PubMed, Web of Science, Scopus and Cochrane Library. Two searches were performed in each database with the following simplified search protocols adapted to each database: (strongyloides OR strongyloidiasis) AND diabetes AND (hyperinfection OR disseminated strongyloidiasis); (strongyloides OR strongyloidiasis) AND immunocompetent.

We included case reports and case report series written in English, German and Romanian. We excluded cases in which known evident immunosuppression factors previously associated with severe strongyloidiasis were presented [10,38,39,40]. 

Severe strongyloidiasis was defined as an increased larval burden with elevated severity of infection (hyperinfection syndrome) or dissemination of larvae to other sites than the gastrointestinal and pulmonary tracts (disseminated strongyloidiasis). Where available, the diagnosis employed by the original authors was maintained. References of included studies were also screened, and relevant studies were included in the review.

The goal of this review was to evaluate the frequency of reported cases of severe strongyloidiasis in patients with diabetes and to examine the extent to which diabetes can be considered a risk factor for the development of hyperinfection or disseminated disease.

To the best of our knowledge, this is the first review to evaluate the relationship between diabetes and severe strongyloidiasis.

### 4.2. Results

The combined searches yielded 239 studies. After the removal of duplicates, the studies were evaluated by two researchers (V.S.N. and I.A.C.), and disputes were resolved through oversight from an expert opinion (C.C.). A total of nine studies were included in the review, with a total of nine cases (six from the combined searches, three from other sources). Taking our case into consideration, a total of 10 cases were included in the review. The results are presented in Table 1.

A few cases were excluded due to the high probability of bias on account of unclear or missing data regarding immunosuppression or the diagnosis of severe strongyloidiasis: local corticosteroid injections [57], multiple myeloma in remission and corticosteroid treatment after hospital admission [58], possible untreated rheumatoid arthritis and short corticosteroid treatment after hospital admission [59], unclear corticoid treatment for asthma and emphysema [60], unclear asthma treatment [61], previous single-dose corticoid treatment [62], previously undiagnosed diabetes and short corticoid treatment after hospital admission [63], possible corticosteroid treatment and partial gastrectomy surgery [64], unclear severe strongyloidiasis diagnosis [65,66].

The majority of included cases presented male patients (n = 9, 90%) with a median age of 59 years (range 31–82 years). A relatively high mortality rate was observed (n = 4, 40%). In most cases, including the case presented by us, the antiparasitic treatment was delayed [50,51,52,53,54,55] (n = 7, 70%). Only one case reported an early treatment [31]. Of the four cases that reported death as the endpoint, treatment was delayed in two cases [50,52], started early in one case [31] and not reported in another [49]. Most cases were treated with ivermectin alone (n = 6, 60%), but albendazole, mebendazole and thiabendazole were also used. A combined treatment regimen with albendazole and ivermectin or mebendazole and thiabendazole was used in two cases (20%). Diagnosis was performed in all cases through microscopic techniques from stool or other samples. Biopsies from the gastrointestinal tract were used alone or in combination with another sample in less than half of the presented cases (n = 4, 40%). Molecular-based techniques or serology were not employed in the diagnosis. Eosinophil count was not reported in one case (10%), and eosinophilia was present in only two cases (n = 2, 20%). One case (10%) highlighted the presence of peripheral bandemia. Only one case was from Europe (10%).

Though the overwhelming majority of severe strongyloidiasis cases occur in immunosuppressed individuals [30,32], a few cases have also been reported in immunocompetent patients [50].

Compared to our review, a lower mortality rate has been reported in immunocompetent patients [50], and consequently, a higher mortality rate has been reported in immunocompromised individuals [30,32]. Furthermore, patients with diabetes seem to present relatively similar rates of eosinophilia as those observed in immunosuppressed patients [30,32]. A detailed comparison of severe strongyloidiasis characteristics in these patient groups is presented in Table 2.

Based on the limited available data, severe strongyloidiasis in patients with diabetes seems to present a higher mortality and lower eosinophilia rate as opposed to cases in immunocompetent patients. Though the lower eosinophilia rates seem to suggest a closer resemblance to cases seen in immunosuppressed patients, the observed mortality rate seen in diabetic patients seems to be in between that observed in immunocompetent and immunosuppressed individuals.

### 4.3. Limitations

Our review presents several limitations, including a relatively low number of cases and a lack of distinction between controlled and uncontrolled diabetes. Additionally, the comparison with other reviews might be biased by multiple factors: a low number of cases in some reviews, an overlap of case reports between reviews, the inclusion of a limited number of patients without immunosuppression in the reviews by Geri et al. [32] and Buonfrate et al. [30], the possible use of different case definitions for hyperinfection syndrome and disseminated strongyloidiasis. Thus, definitive conclusions are hard to draw based on the limited available data.

Even though there are limited cases available at this moment, we believe that elucidating a potential link between severe strongyloidiasis and diabetes is important. If such a link exists, this would present clinical significance, in particular in light of the increasing prevalence of diabetes worldwide coupled with the aging of the population and steady increase in travel and migration.

## 5. Conclusions

Early diagnosis through multiple diagnostic techniques prompted by a high index of clinical suspicion is paramount in decreasing the mortality rate of severe strongyloidiasis. We believe that the case and review presented by us will provide a helpful reminder to include severe strongyloidiasis in the list of differential diagnoses even in non-endemic countries and in the absence of classical immunosuppression factors.

The extent to which diabetes can be considered a risk factor for severe strongyloidiasis remains an open question at this moment. The limited available evidence seems to point out that diabetes can be taken into consideration as a risk factor for severe strongyloidiasis. However, further research is needed in order to clarify this relationship.

## Figures and Tables

**Table 1:** Severe strongyloidiasis (hyperinfection syndrome and disseminated disease) in individuals with diabetes.

Reference	Patient Data	Country of Origin/Recent Trips or Immigration	Eosinophilia	Comorbidities	Severe Strongyloidiasis	Diagnosis	Treatment	Complications	Outcome
Kishimoto et al., 2008 [49]	82, M	Japan	N/A	-	H/D	Microscopy (stool, sputum)	ivermectin	Small bowel obstruction, meningitis, pneumonia	Died
Chan et al., 2018 [50]	72, M	Australia/Southeast Asia	No	Peripheral vascular disease, dyslipidemia, hypertension, gastro-esophageal reflux, asthma, recent laparotomy for inguinal hernia	H	Microscopy (mesenteric biopsy, tracheal aspirate)	ivermectin, 200 mcg/kg daily	Multi-organ failure	Died
Current case	71, M	Romania	No	Hypertension, benign prostatic hyperplasia, colonic diverticular uncomplicated disease	H	Microscopy (stool, duodenal biopsy)	albendazole, 400 mg twice daily for 10 days ivermectin, 200 mcg/kg for 3 days	Partial duodenal obstruction	Survived
Emad et al., 1999 [51]	61, M	Iran	Yes	-	H/D	Microscopy (bronchial washing)	thiabendazole, 1.5 g twice/day for 21 days	Eosinophilic pleural effusion	Survived
Myint et al., 2017 [52]	60, M	USA/El Salvador	No Peripheral bandemia	Hypertension, obesity, alcohol consumption (stopped 8 months prior)	H	Microscopy (stool)	ivermectin	Duodenitis, small bowel obstruction, pulmonary hemorrhage and oedema, cardiac arrest	Died
Murali et al., 2010 [53]	58, M	India	No	Total laryngectomy due to carcinoma (4 years prior)	D	Microscopy (pericardial fluid)	ivermectin, 12 mg for 2 days	Pericardial effusion	Survived
Lam et al., 2006 [31]	57, M	Hong Kong	No	-	D	Microscopy (gastric biopsy)	mebendazole, 100 mg two times a day thiabendazole	Multiple organ involvement, diabetic ketoacidosis	Died
St. Cyr et al., 2013 [54]	51, F	USA/Laos	No	Hypertension, hyperlipidemia, gastroesophageal reflux disease	H	Microscopy (stool)	ivermectin, 200 mcg/kg for 2 days	Bloodstream infection due to Shiga toxin producing *E. coli*	Survived
Sridhara et al., 2008 [55]	45, M	USA/Ghana	Yes	Colon polyps, lactose intolerance, internal hemorrhoids	H	Microscopy (colon biopsy)	ivermectin, 15 mg/day for 5 days	Pancolitis	Survived
Tiwari et al., 2012 [56]	31, M	India	No	- *	H	Microscopy (stool)	albendazole, 10 mg/kg/day for 7 days	-	Survived

H = hyperinfection syndrome, D = disseminated strongyloidiasis, N/A = not available; * = recently diagnosed diabetes (4 months prior). The included cases did not present any discernible immunosuppression factors other than diabetes. No distinction was made between controlled or uncontrolled diabetes due to missing information in most cases.

**Table 2:** Comparison of severe strongyloidiasis mortality and eosinophilia in immunocompetent, immunosuppressed and patients with diabetes.

Immune Status	Immunocompetent	Diabetes	Immunosuppressed	
**Reference**	Chan et al. [50]	Current review	Geri et al. [32]	Buonfrate et al. [30]
**Sample size**	n = 9	n = 10	n = 133	n = 244
**Male gender**	88.9%	90%	72.2%	N/A
**Mortality**	22.2%	40%	60.3%	62.7%
**Eosinophilia**	66.7%	20%	34.3%	22.5%

## Data Availability

All data are contained within the article.
